# Characterization of plasma proteins in children of different *Mycobacterium tuberculosis* infection status using label-free quantitative proteomics

**DOI:** 10.18632/oncotarget.21179

**Published:** 2017-09-23

**Authors:** Jieqiong Li, Lin Sun, Fang Xu, Jing Xiao, Weiwei Jiao, Hui Qi, Chen Shen, Adong Shen

**Affiliations:** ^1^ Beijing Key Laboratory of Pediatric Respiratory Infection Diseases, Beijing Pediatric Research Institute, Beijing Children’s Hospital, Capital Medical University, National Center for Children’s Health, Beijing, China; ^2^ National Clinical Research Center for Respiratory Diseases, Beijing, China; ^3^ National Key Discipline of Pediatrics, Capital Medical University, Beijing, China; ^4^ Key Laboratory of Major Diseases in Children, Ministry of Education, Beijing, China

**Keywords:** plasma proteins, active tuberculosis (ATB), latent TB infection (LTBI), children, label-free quantitative proteomics

## Abstract

Tuberculosis (TB), caused by *Mycobacterium tuberculosis* (MTB), is an infectious disease found worldwide. Children infected with MTB are more likely to progress to active TB (ATB); however, the molecular mechanism behind this process has long been a mystery. We employed the label-free quantitative proteomic technology to identify and characterize differences in plasma proteins between ATB and latent TB infection (LTBI) in children. To detect differences that are indicative of MTB infection, we first selected proteins whose expressions were markedly different between the ATB and LTBI groups and the control groups (inflammatory disease control (IDC) and healthy control (HC) groups). A total of 521 proteins differed (> 1.5-fold or < 0.6-fold) in the LTBI group, and 318 proteins in the ATB group when compared with the control groups. Of these, 49 overlapping proteins were differentially expressed between LTBI and ATB. Gene Ontology (GO) analysis revealed most proteins had a cellular and organelle distribution. The MTB infection status was mainly related to differences in binding, cellular and metabolic processes. XRCC4, PCF11, SEMA4A and ATP11A were selected and further verified by qPCR and western blot. At the mRNA level, the expression of XRCC4, PCF11and SEMA4A presented an increased trend in ATB group compare with LTBI. At the protein level, the expression of all these proteins by western blot in ATB/LTBI was consistent with the trends from proteomic detection. Our results provide important data for future mechanism studies and biomarker selection for MTB infection in children.

## INTRODUCTION

Tuberculosis (TB), caused by *Mycobacterium tuberculosis* (MTB), continues to be a serious infectious disease worldwide [1, 2]. It is estimated that most active TB (ATB) cases originate from an initial latent TB infection (LTBI), a state without any clinical symptoms, radiological abnormality, and microbiological evidence. In contrast to adults, young children infected with MTB are more likely to progress to ATB within the first year of primary infection; however, the molecular mechanisms behind this have long been a mystery [3].

MTB infection alters the expression of TB-associated proteins, which are released into the bloodstream through different pathways [4]. Thus, analysis of TB-associated proteins in patients with different MTB infection status might reflect the mechanism of MTB infection and progression. Plasma proteins, such as cytokines, extracellular secretory proteins, and other soluble factors involved in the immune system, are reported to be associated with the pathogenesis of infectious disease [5]. For instance, Shuxian Li *et al.* revealed that the proteins secreted by A549 cells were associated with the infection of *Mycoplasma pneumoniae* [6]. Dandan Xu and her colleagues found that the level of serum proteins (S100A9, SOD3, and MMP9) in ATB reflected the immune response to the MTB infection [5]. Together, the above information implies an important role for proteins in host-pathogen interactions.

Recently, advances in comprehensive analytical techniques, such as proteomics, make the analysis of all plasma proteins possible [7]. Proteomic analysis using the label-free quantitative protocol is a novel approach for high throughput analysis, which provides for a rapid comparison of proteins. Given that a specific mixture of plasma proteins has distinctive characteristics, this technique has been used to investigate the proteomic patterns in many diseases, including infectious diseases [8–11], vascular diseases [12–14] and cancer [15]. Thus, analysis of the expression of TB-associated proteins in plasma could provide a better understanding of conversion from LTBI to ATB in children.

Therefore, this study aimed to characterize the plasma proteins in children of different MTB infection status by label-free quantitative proteomics. We observed that 49 proteins were differentially-expressed in the LTBI and ATB groups, and a subset was validated using quantitative real-time PCR (qPCR) and western blotting. Our results will provide insight into the mechanism of MTB infection and progression, as well as potential biomarker selection for the diagnosis of ATB in children.

## RESULTS

### Clinical characteristics

The demographic characteristics of participants showed no obviously differences between groups (Table 1; age, *P* = 0.625; gender, *P* = 0.318). Among the 19 children in the ATB group, 12 had pulmonary TB, three had tubercular meningitis, one had bone tuberculosis and three had systemic tuberculosis. Among the 17 children in the IDC group, 10 had pneumonia (including both bacterial and viral infections), and seven children had upper respiratory tract infections. Furthermore, the children in inflammatory disease control (IDC) and healthy control (HC) groups had negative interferon gamma release assay (IGRAs) and tuberculin skin test (TST) results, without a history of ATB. All the subjects were vaccinated with BCG.

**Table 1:** Demographic characteristic of the participants

Characteristics	ATB	LTBI	IDC	HC
Sample size	19	16	17	20
Age (years)^a^	6.8 ± 5.1	7.2 ± 3.2	6.9 ± 4.7	7.4 ± 3.5
Age range (years)	0.2-16	3-11	0.8-15.3	4-14
Gender (Male/Female)	10/19	8/16	9/17	9/20
BCG vaccine	19/19	16/16	17/17	20/20

^a^Data are presented as the mean ± SD.

### Label-free quantitative proteomics analysis of the differences between ATB and LTBI

As respiratory infections are often present in children who have ATB or LTBI, both IDC and HC groups were included in this proteomics analysis. Based on the high throughput proteomics data, a total of 2170 proteins were identified. The plasma, pooled for each group was divided into 3 samples and each liquid chromatography-mass/mass spectrometry (LC-MS/MS) analysis was repeated 3 times.

**Figure 1:**
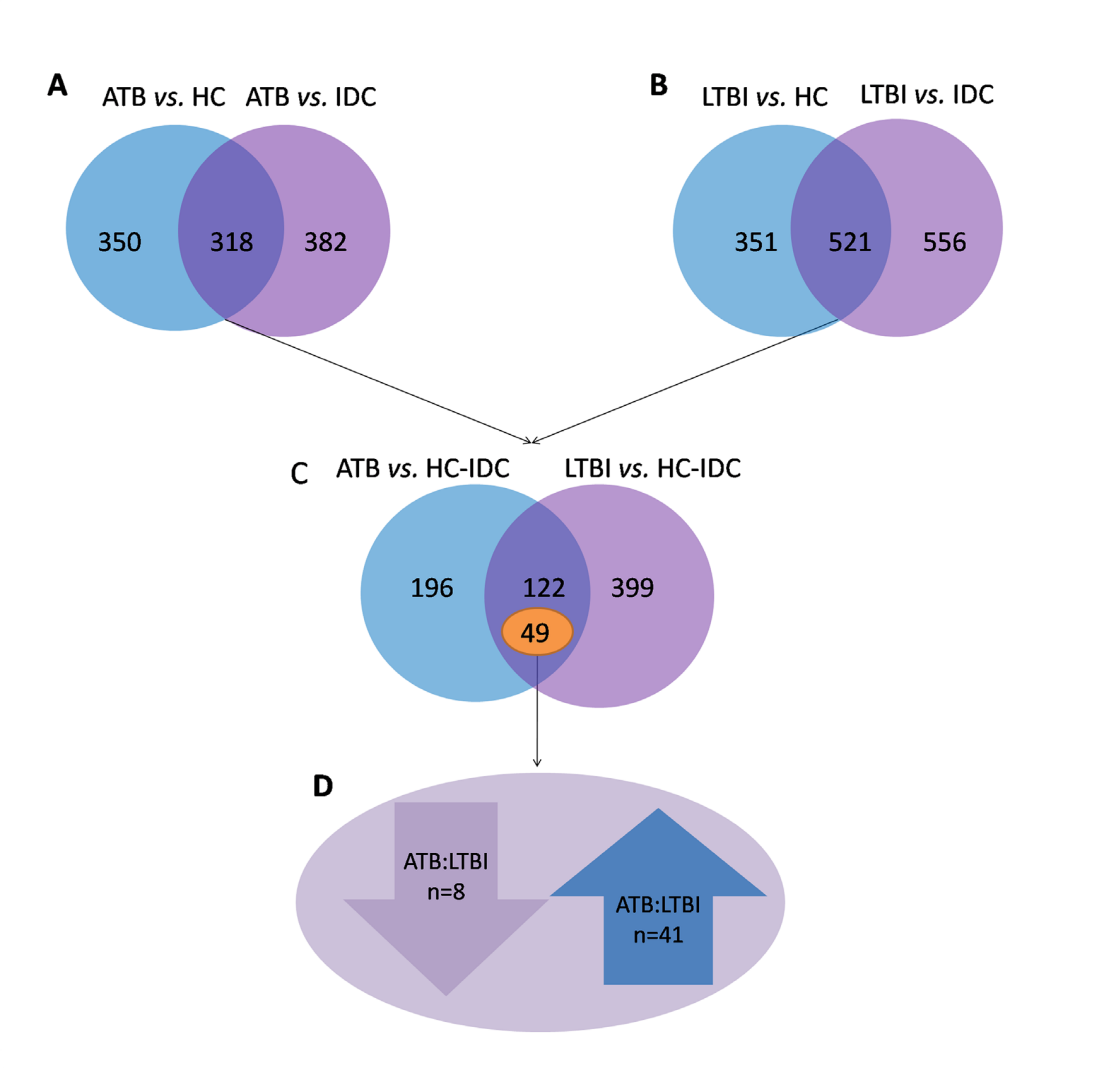
Different proteins selected through LC-MS/MS analysis **(A)** 318 proteins were significantly different in ATB subjects compared with the HC and IDC groups. **(B)** 521 proteins were significantly different in LTBI subjects compared with the HC and IDC groups. **(C)** Among these protein, 49 proteins were markedly different between ATB and LTBI. **(D)** 41 proteins were up-regulated (>1.5 fold) and 8 proteins were down-regulated (< 0.6-fold) in the ATB group compared with the LTBI group.

To detect the proteins that are indicative of MTB infection, we first selected specific proteins whose expression was markedly different between the ATB and control (IDC and HC) groups, and between the LTBI groups and control (IDC and HC) groups. As shown in Figure 1, a total of 318 proteins differed in the ATB group, and 521 proteins in the LTBI group (> 1.5 fold or < 0.6 fold) when compared with the HC and IDC groups. Of these proteins associated with MTB infection, 49 proteins were differentially expressed between ATB and LTBI. Specifically, 41 proteins were up-regulated (> 1.5 fold) and 8 proteins were down-regulated (< 0.6 fold) in the ATB group compared with the LTBI group (Table 2).

**Table 2:** Differentially expressed proteins identified following LC-MS/MS of ATB and LTBI plasma fractions

Gi number	Protein name	Gene	Uniprot Identifier	mass	pI	ATB:LTBI
gi|282165800	tight junction protein ZO-2 isoform 5	*TJP2*	B7Z2R3	115.10	6.30	↓
gi|6912752	zinc finger protein 281	ZNF281	Q9Y2X9	96.90	9.50	↓
gi|122114658	hypothetical protein LOC23053	ZSWIM8	A7E2V4	197.70	6.40	↓
gi|310110387	PREDICTED: hypothetical protein LOC100508795	-	-	13.60	10.80	↓
gi|296317364	testis-specific serine/threonine-protein kinase 4 isoform 1	TSSK4	Q6SA08	38.40	9.60	↓
gi|113722125	type II inositol-1,4,5-trisphosphate 5-phosphatase precursor	INPP5B	P32019	103.90	5.10	↓
gi|40556393	protein Jade-1 long isoform	JADE1	Q6IE81	95.50	9.20	↓
gi|20149540	protein-lysine 6-oxidase isoform 1 preproprotein	LOX	P28300	46.90	9.10	↓
gi|24308211	integrator complex subunit 2	INTS2	Q9H0H0	134.30	5.70	↑
gi|105990532	apolipoprotein B-100 precursor	APOB	P04114	515.20	6.60	↑
gi|140161498	microtubule-associated tumor suppressor candidate 2 isoform a	MTUS2	Q5JR59	151.10	6.30	↑
gi|33356174	pinin	PNN	Q9H307	81.60	6.80	↑
gi|169169660	PREDICTED: hypothetical protein LOC100131673	-	-	50.80	9.40	↑
gi|110611228	utrophin	UTRN	P46939	394.20	5.10	↑
gi|28872812	MORC family CW-type zinc finger protein 3	MORC3	Q14149	107.00	5.30	↑
gi|32307152	oxytocin receptor	OXTR	P30559	42.70	10.90	↑
gi|38016914	SAM domain and HD domain-containing protein 1	SAMHD1	Q9Y3Z3	72.20	6.80	↑
gi|21361912	dnaJ homolog subfamily C member 1 precursor	DNAJC1	Q96KC8	63.80	9.40	↑
gi|157266264	pleckstrin homology-like domain family B member 3	PHLDB3	Q6NSJ2	71.90	6.10	↑
gi|4504445	heterogeneous nuclear ribonucleoprotein A1 isoform a	HNRNPA1	P09651	34.20	9.70	↑
gi|148806908	fibronectin type III domain-containing protein 1	FNDC1	Q4ZHG4	205.40	9.80	↑
gi|303304991	centrosomal protein of 152 kDa isoform 1	CEP152	O94986	195.50	5.30	↑
gi|222136639	C-1-tetrahydrofolate synthase, cytoplasmic	MTHFD1	P11586	101.50	7.00	↑
gi|59710085	hypothetical protein LOC146562	C16orf71	Q8IYS4	55.60	4.70	↑
gi|291045249	collagen alpha-1(XIII) chain isoform 19	COL13A1	Q5TAT6	59.40	9.90	↑
gi|54112117	splicing factor 3B subunit 1 isoform 1	SF3B1	O75533	145.70	6.70	↑
gi|31377667	lon protease homolog 2, peroxisomal	LONP2	Q86WA8	94.60	7.00	↑
gi|7949031	cytochrome P450 2B6	CYP2B6	P20813	56.20	9.10	↑
gi|51702222	protein SPT2 homolog	SPTY2D1	Q68D10	75.60	10.30	↑
gi|150421681	probable phospholipid-transporting ATPase IH isoform b	ATP11A	P98196	135.80	6.30	↑
gi|55741447	pleckstrin homology domain-containing family H member 1	PLEKHH1	Q9ULM0	151.10	9.00	↑
gi|33620745	pre-mRNA cleavage complex 2 protein Pcf11	PCF11	O94913	172.90	9.30	↑
gi|38027923	COP9 signalosome complex subunit 5	COPS5	Q92905	37.60	6.10	↑
gi|45827701	protein dopey-2	DOPEY2	Q9Y3R5	258.10	5.90	↑
gi|118421085	treslin	TICRR	Q7Z2Z1	210.70	9.80	↑
gi|113416493	PREDICTED: putative TAF11-like protein ENSP00000332601-like	-	-	21.60	7.70	↑
gi|159032029	sentrin-specific protease 5	SENP5	Q96HI0	86.60	10.10	↑
gi|28827774	dual specificity tyrosine-phosphorylation-regulated kinase 4	DYRK4	Q9NR20	59.60	9.70	↑
gi|40804748	LIM and senescent cell antigen-like-containing domain protein 2 isoform 2	LIMS2	Q7Z4I7	41.50	10.10	↑
gi|5454058	CMP-N-acetylneuraminate-beta-galactosamide-alpha-2,3-sialyltransferase 4	ST3GAL4	Q11206	37.40	10.00	↑
gi|19923437	GTP:AMP phosphotransferase, mitochondrial	AK3	Q9UIJ7	25.50	9.50	↑
gi|50659100	inactive serine protease PAMR1 isoform b	PAMR1	Q6UXH9	80.10	8.80	↑
gi|4507945	DNA repair protein XRCC4 isoform 1	XRCC4	Q13426	38.00	4.80	↑
gi|164519084	rab GTPase-activating protein 1	RABGAP1	Q9Y3P9	121.70	5.00	↑
gi|98986457	host cell factor 1	HCFC1	P51610	208.60	7.90	↑
gi|310123245	PREDICTED: Golgin subfamily A member 8-like protein 2-like, partial	-	-	62.60	6.20	↑
gi|300863076	semaphorin-4A isoform 2	SEMA4A	Q9H3S1	69.10	6.30	↑
gi|310113571	PREDICTED: hypothetical protein LOC100508805	-	-	68.60	6.60	↑
gi|46409310	zinc finger protein 467	ZNF467	Q7Z7K2	65.10	11.00	↑

**Figure 2:**
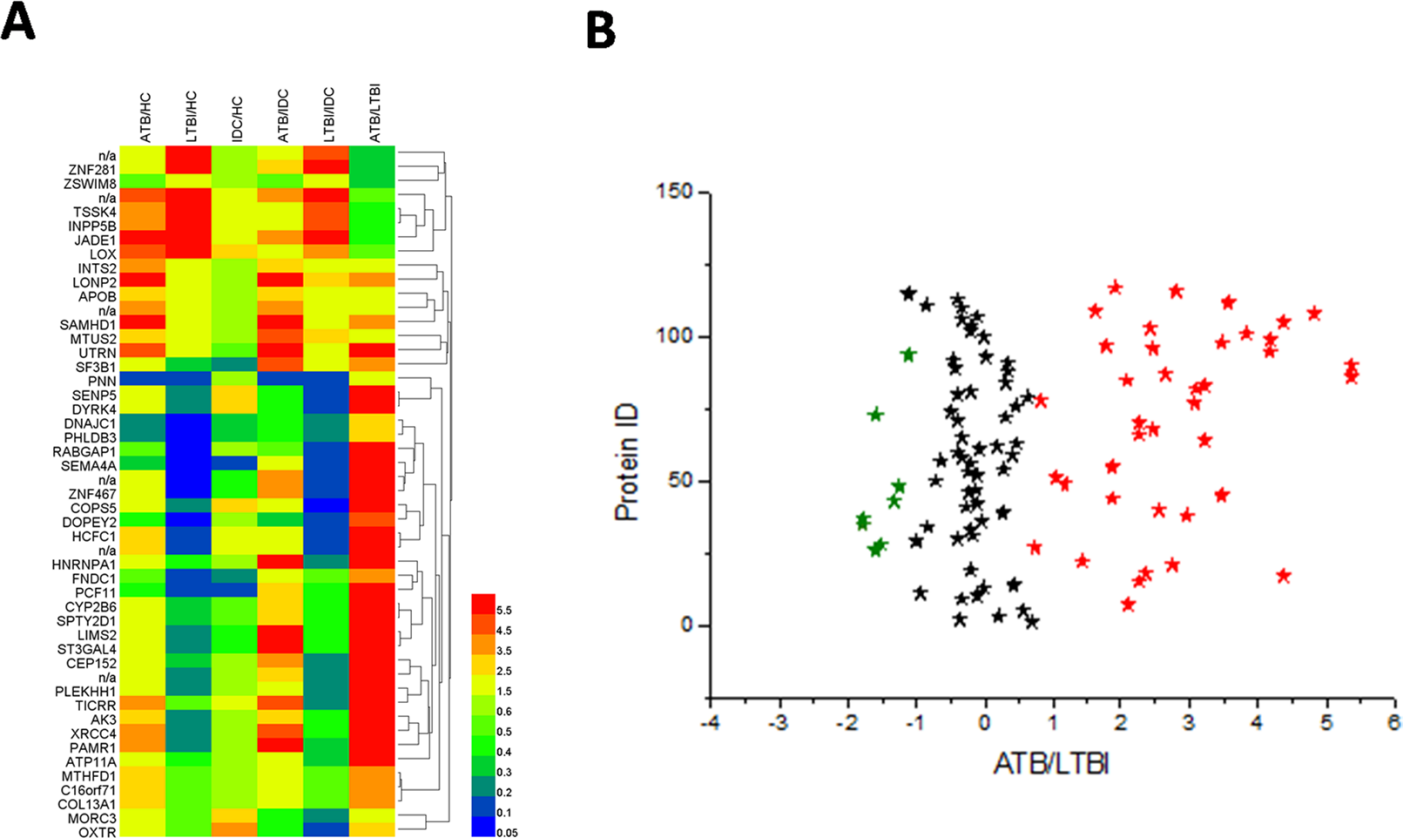
Heat map and volcano plot of the 49 identified proteins **(A)** The most striking area of up-regulation in ATB patients is seen in the region where a series of protein peaks are shown in red. **(B)** Important features selected by volcano plot.

To reveal the relationship between the 49 different proteins, a hierarchical clustering based on Pearson correlation of variances was applied using R studio. Figure 2A depicts hierarchical clustering of the 49 identified proteins, where an increasingred color indicates increasing protein expression levels. Thus, the most prominent area of up-regulation in ATB subjects was seen in the region where these protein peaks are shown in red. Furthermore, important features were selected by a volcano plot (Figure 2B).

### Gene ontology analysis of the identified proteins

**Figure 3:**
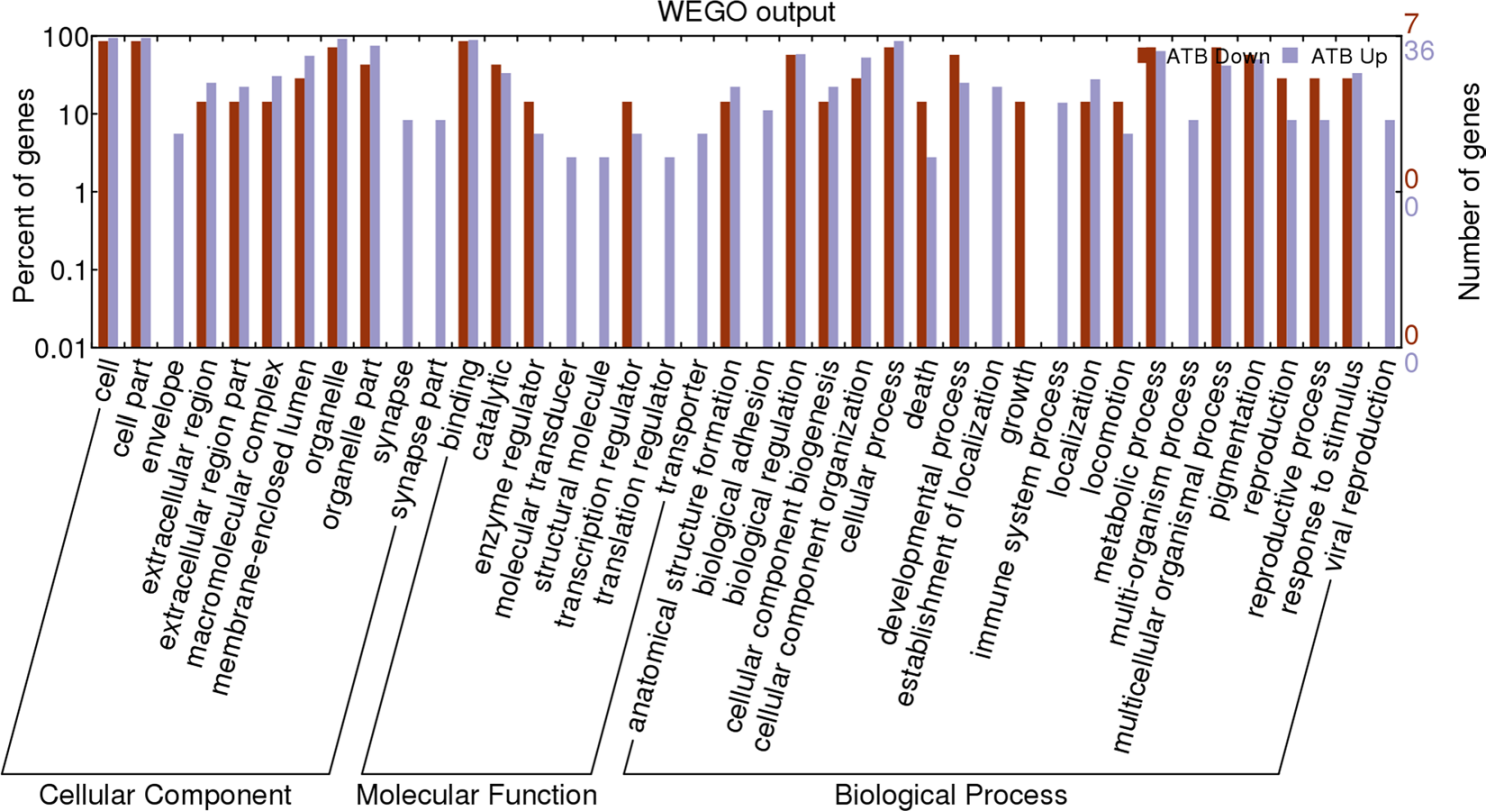
Web Gene Ontology Annotation Plot (WEGO) classification of differentially expressed proteins by label-free quantitative proteomics experiments between ATB and LTBI The differentially expressed proteins are grouped into three hierarchically structured terms: biological process, cellular component, and molecular function.

To further understand the functions of these identified proteins, we classified the 49 differential proteins between LTBI and ATB by their Gene Ontology (GO) categories, including cellular component (CC), molecular function (MF), and biological process (BP) using the Web Gene Ontology Annotation Plotting (WEGO, Figure 3, Supplementary Table 1 and 2). According to the analysis of CC, the majority of the proteins had a cell and organelle distribution. According to the analysis of BP, the primary functions were cellular processes, metabolic processes and biological regulation; while others were related to cellular component organization, pigmentation, multicellular organismal processes and so on. According to the analysis of MF, these proteins could be classified into eight categories as follows: molecular transducer activity, translation regulator activity, enzyme regulator activity, transcription regulator activity, structural molecule activity, catalytic activity, binding, transporter activity. Among them, a large proportion had binding as a molecular function. To identify the most important proteins involved in the conversion from LTBI to ATB, proteins that were obviously up or down-regulated should be categorized into many different functional groups.

### KEGG enrichment analysis of the identified proteins

**Figure 4:**
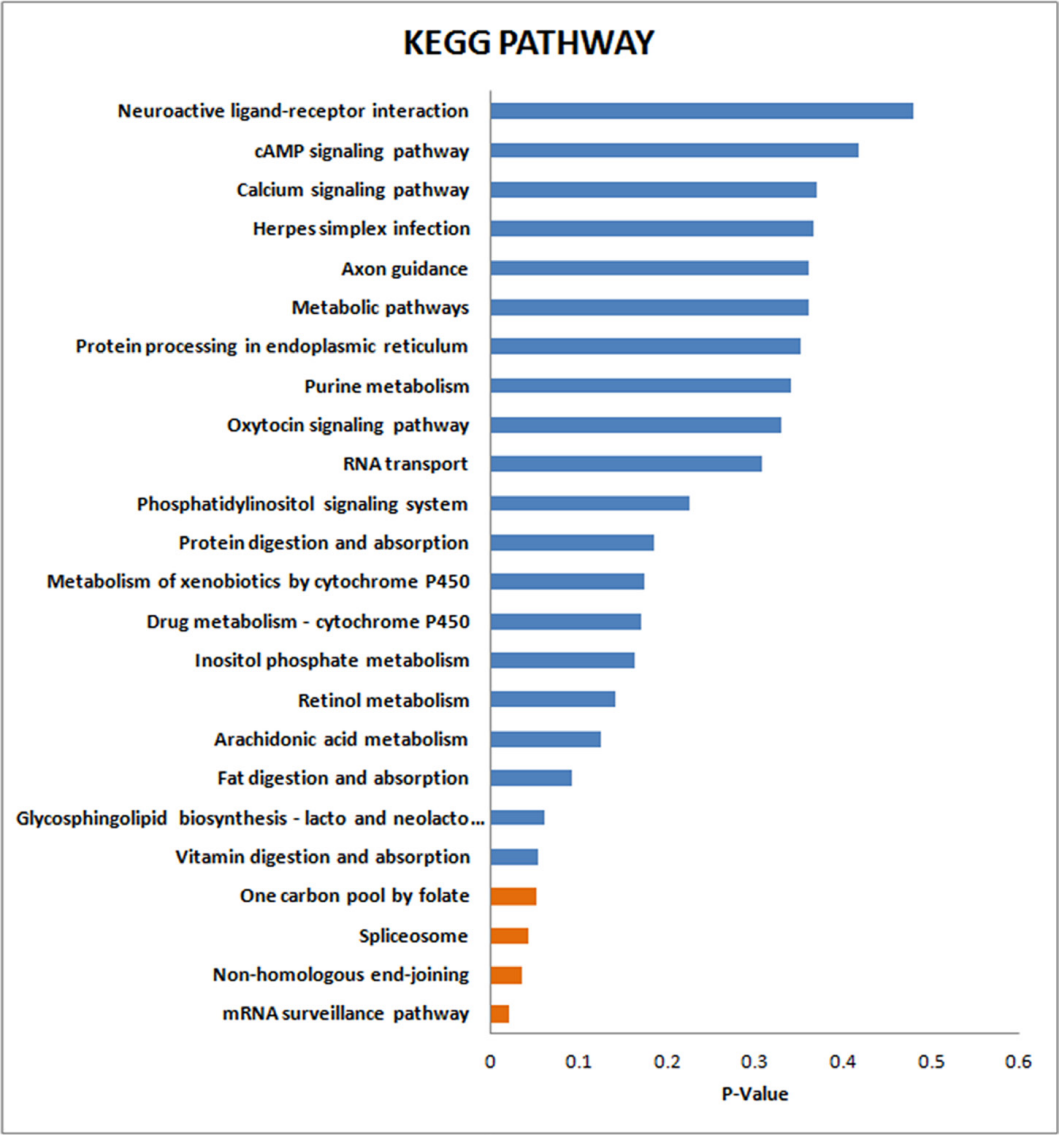
KEGG enrichment analysis of the differentially expressed proteins

KEGG enrichment analysis was then implemented to test enrichment of the proteomics pathways between the LTBI and ATB groups. The results indicated that mRNA surveillance pathway (*P* = 0.018), Non-homologous end-joining (*P* = 0.034) and Spliceosome (*P* = 0.040), One carbon pool by folate (*P* = 0.049) were significantly associated with the conversion from LTBI to ATB (Figure 4 and Supplementary Table 3).

### Validation of proteomic results

Proteins identified as differentially expressed in ATB and LTBI patients were validated using qPCR and western blotting. Among the 49 different proteins, four (XRCC4, PCF11, SEMA4A and ATP11A) were selected for further verification. The selection of the proteins for the verification stage was based on the following criteria: (1) high fold change; (2) being representative of the 49 different proteins; (3) *P* value of <0.05 in KEGG analysis (XRCC4 and PCF11) or having biological functions associated with immunity and other infectious diseases (SEMA4A and ATP11A). Although these four proteins were mainly distributed in cellular parts, their functions were different. Most proteins different between the ATB and LTBI groups were related to binding, cellular and metabolic process and these four selected proteins were associated with these processes. Firstly, the four selected proteins all were also participate in the binding processes, including nucleic acid binding and protein binding. Additionally, XCRR4 and PCF11 had an effect on metabolic process while SEMA4A and ATP11A were related to the cellular process. Thus, these four proteins, being representative of the 49 different proteins, reflected the different status of MTB infection.

**Figure 5:**
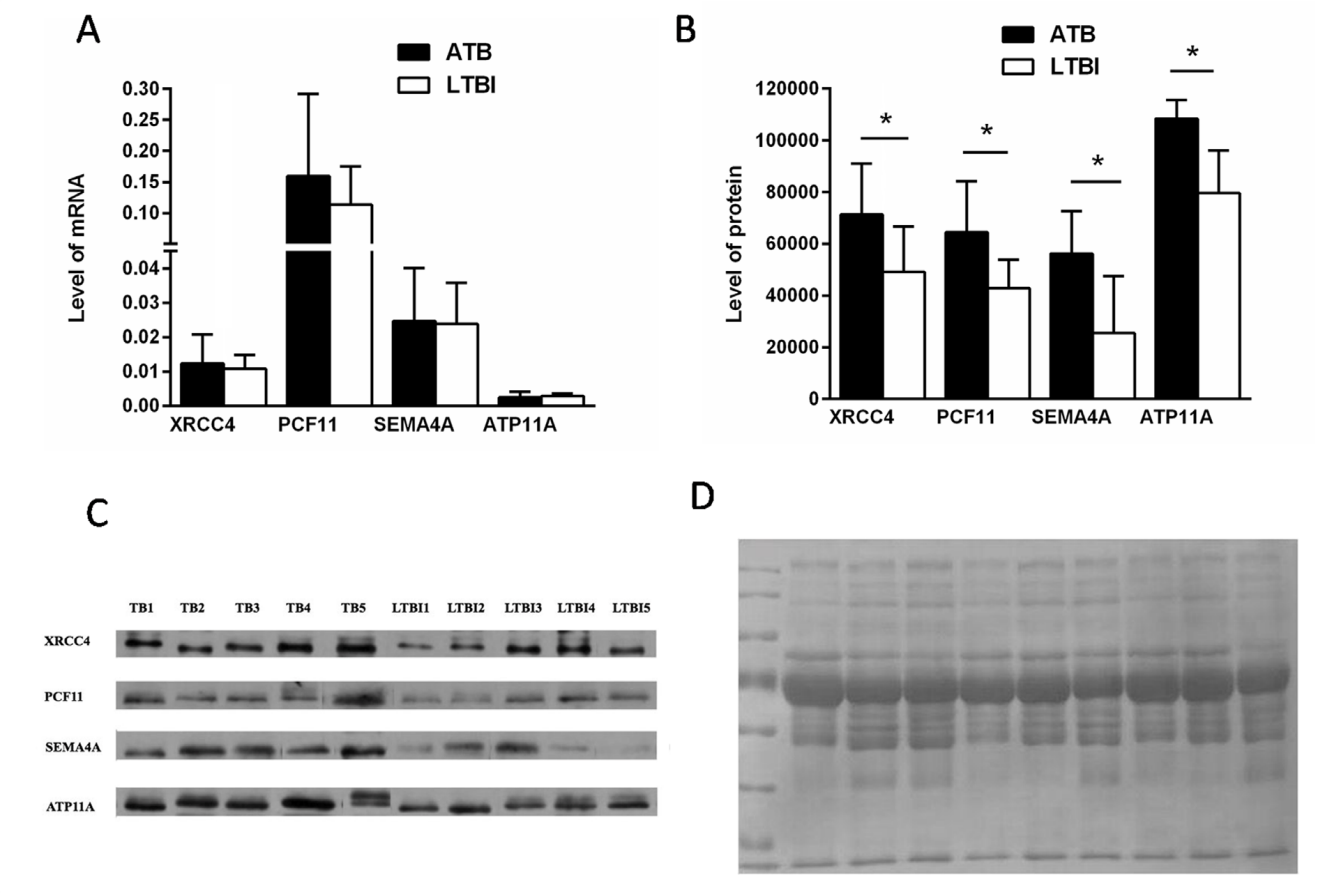
Verification of up- or down-regulated proteins between ATB and LTBI **(A)** RT-PCR analysis data of four selected proteins in the PBMCs of ATB patients compared with that of LTBI patients (n_ATB_ = 28, n_LTBI_ = 18); data are presented as means ± SD. **(B)** The average signals of ATB and LTBI patients group (*P* < 0.05, n = 5 per group); data are presented as means ± SD. **(C)** Western blot analysis of the four selected proteins from ATB and LTBI subjects (n=5 per group). **(D)** Total protein staining by Coomassie Blue employed as the loading control.

At the mRNA level, the expression of XRCC4, PCF11and SEMA4A presented an increased trend in ATB group compare with that in LTBI and this agreed with the proteomic results. The ratio of ATP11A was apparently inconsistent with the proteomic data (Figure 5A). The mRNA result implies that regulation is occurring at the point of protein translation, not gene transcription. At the protein level, our results demonstrated that the plasma levels of XRCC4, PCF11, SEMA4A and ATP11A in the ATB group were significantly higher than the LTBI group (all *P* < 0.05; Figure 5B). Representative western blots are shown in Figure 5C. These ratios were consistent with the trends of proteomics results. Total protein staining by Coomassie Blue employed as the loading control (Figure 5D).

## DISCUSSION

It is generally known that the immune response induced by MTB infection influences the pathogenic mechanisms and protein expression [16]. Specifically, TB-associated proteins appear in the circulation by a variety of mechanisms, including direct secretion of proteins, stimulated production of reactive proteins, or production of proteins during the infection of the MTB [17, 18]. Accurate detection TB-associated proteins lead to a better understanding of the mechanism of MTB infection and progression.

Plasma proteins including cytokines, extracellular secretory proteins, growth factors, and other soluble factors, contribute to many physiological processes. Recently, researchers have demonstrated that proteins are also significantly associated with different diseases. For instance, the secretion of adiponectin is associated with the development of many cardiovascular related diseases [19]. Similarly, plasma proteins also have profound effects on a variety of infectious diseases. For example, during herpes simplex virus 1 (HSV-1) infection, increased IFN-induced protein expression is reported to play an essential role in extracellular antiviral functions [20]. Similarly, the secretion of proteins may increase the number of CD4^+^ T cells [21]. Taken together, the above data show the importance of plasma proteins during pathogen infection.

This study demonstrated a difference in plasma protein pattern in MTB infection in children. As respiratory infections are often present in children who have ATB or LTBI, we included both IDC and HC groups in this proteomic analysis. To detect differences that are indicative of MTB infection, we first selected specific proteins whose expression was markedly different in ATB or LTBI groups compared with controls (the IDC and HC groups). Among these proteins, 49 were differentially expressed between LTBI and ATB. These proteins were further performed by volcano plot. The traditional volcano plot is a combination of fold change and t-tests. Note, for unpaired samples, the x-axis is log (FC). Y-axis is -log10 (*P*-value) for both cases, and can be based on raw or FDR adjusted *P* values. However, our data alignment strategy is: disease control and normal control, respectively, compared with ATB and LTBI, followed look at the difference between the significant difference between the part of ATB and LTBI. In our data, the three biological repetitions and three technical repetitions combined after the comparison, so cannot achieve the traditional meaning of the volcano show. Figure 2B just showed the different proteins between ATB and LTBI.

These plasma proteins were further subjected to a GO enrichment analysis which clustered proteins based on three defined ontology terms: GO-CC, GO-BP and GO-MF [22]. Here, we observed that most of these proteins were present in the isolated cell or organelle fraction. Similarly, released intracellular proteins were detected in MTB-infected plasma, suggesting the importance of these associated proteins at the lesions created by MTB infection. In addition, other identified proteins were associated with protein binding. These results demonstrate that the proteins affecting the MTB infection status were related to DNA and protein binding, cellular and metabolic processes.

Among the 49 differentially expressed proteins, XRCC4, PCF11, SEMA4A and ATP11A were selected for further validation. The above functional analysis demonstrated that most different proteins influencing the MTB infection status were mainly related to binding, cellular and metabolic processes. The four selected proteins were all associated with these processes. It is well known that binding is the first step of the MTB infection and these four selected proteins were all participate in the binding process, including nucleic acid binding and protein binding. Furthermore, MTB infection also changes metabolic processes. XCRR4 and PCF11 influence metabolic processes and were significantly different between the ATB and LTBI groups. According to the GO analysis, SEMA4A and ATP11A, which are related to cellular processes, also had an important role in MTB infection.

The levels of mRNA after MTB infection did not correlate with the proteins level. A similar result demonstrated that when the most induced proteins in human mesenchymal stromal cells (HMSCs) were assayed by qPCR, the results do not match the results detected by MS [23]. This is a limitation of the current study.

The selected proteins have previously been reported to be associated with immunity and other infectious diseases [24–35]. XRCC4 is an important component of non-homologous end-joining [24]. The expression of this gene is associated with many infectious diseases, including HSV-1 [25, 26]. In addition, PCF11serves as a target for regulated transcription and pre-mRNA processing. Recent studies showed that it is also involved in regulated transcription initiation, elongation, termination and alternative processing of pre-mRNA [27–29]. SEMA4A is expressed by dendritic cells, macrophages, and activated T cells [30, 31]. It is involved in co-stimulation in helper T cell proliferation and cytokine production [32, 33]. Higher levels of SEMA4A in ATB group implied the higher T cell activation in MTB infection than in LTBI. ATP11A, a member of P4-ATPases, is a lipid flippase [34]. A previous study demonstrated that lipid flippases are essential to mediate the endotoxin-induced endocytosis of Toll-like receptor 4 in human macrophages. Macrophages play a primary role in TB transmission [35]. MTB usually replicates within macrophages and spreads to pulmonary lymph nodes. Furthermore, lipid flippase is also important to dampen the inflammatory response, implying that ATP11A had an important effect in MTB infection. Our results indicate, for the first time, that the expression of these proteins were also correlated with the conversion from LTBI to ATB in children.

Because of the difficulty of enrolling LTBI pediatric subjects, a limitation of this study is the relatively small sample size used for verification. Thus, a larger sample size and further mechanism analysis should be the focus of future investigations.

In conclusion, we identified 49 differentially expressed plasma proteins related to the differential status of MTB infection by the label-free quantitative method. After analyzing the protein functions and regulatory networks, XRCC4, PCF11, SEMA4A and ATP11A were selected and further verified by qPCR and western blot. Our results will provide important data for molecular mechanism studies and biomarker selection during MTB infection.

## MATERIALS AND METHODS

### Patients and controls

Respiratory infectious diseases overlapping with TB in children are very common. Therefore, to detect the differences that are indicative of MTB infection, IDC and HC were also included in this study.

A total of 72 children (19 ATB, 16 LTBI, 17 IDC and 20 HC) were enrolled at Beijing Children’s Hospital between July 2010 to May 2013. The diagnosis of pediatric ATB was according to the guidelines of the Chinese Medical Association: (1) MTB culture result was positive, or (2) at least one symptom, sign, or radiographic evidence was consistent with ATB, or (3) clinical and radiological improvement was seen following anti-TB chemotherapy [2]. To accurately reflect the MTB infection status, all the ATB sera were collected before anti-TB chemotherapy.

LTBI was defined as (1) positive by both IGRAs and TST, (2) exposure history to a known ATB case, (3) no clinical, radiological and microbiological evidence of active TB [36].

IDC group subjects were enrolled according to their respiratory symptoms and negative IGRAs and TST results (<5mm) to exclude ATB and LTBI. HC group subjects were recruited from children admitted to Beijing Children’s Hospital for physical examination from June 2012 to May 2013. The IDC and HC controls with negative IGRAs and TST results, had not been previously infected with TB, HIV or other infectious diseases. Each group was matched by age, sex, and ethnicity.

At the qPCR verification stage, we utilized the peripheral blood mononuclear cell (PBMC) samples from 28 ATB (18 boys and 10 girls; median age 5.0 years, range 0.4–12 years) and 18 age- and gender-matched LTBI subjects(12 boys and 6 girls; median age 5.6 years, range 0.25–15 years). At the western blot verification stage, the plasma of 5 ATB (3 boys and 2 girls; median age 8.3 years, range 3–12 years) and 5 age- and gender-matched LTBI subjects (2 boys and 3 girls; median age 7.6 years, range 3–11 years) were collected. The diagnosis criteria of pediatric ATB and LTBI outlined above. All the subjects had the positive IGRAs and TST results.

The study was approved by the Ethics Committee of Beijing Children’s Hospital. All the methods and experimental protocols in this research were performed according to the approved protocols and the Ethics Committees’ existing guidelines. The parents of all children provided written, informed consent prior to their enrollment in the study.

### Protein extraction and digest

Human plasma samples were pooled for each group and divided into 3 aliquots. Albumin, IgG, IgA, antitrypsin, transferrin and haptoglobin were removed by the Agilent Multiple Affinity Removal Column (Multiple Affinity Removal Column, 4.6 mm × 50 mm; Agilent Technologies, Palo Alto, CA, USA). After being resuspended with PBS (50 μL), the samples were centrifuged at 10,000*g* for 30 min in 4°C and resuspended with 100 μL lysis buffer (7 M urea, 2 M thiourea). Total proteins were extracted by ultrasonic sonication and precipitated with trichloroacetic acid (TCA) for 30 min on ice before centrifuging at 40,000*g* for 30 min. Next, the protein concentration was adjusted with 50 mM NH_4_HCO_3_ to a final concentration of 0.5mg/mL and then mixed with DTT (5μl, 1mol/L) and incubated at 37°C for 60 min. After incubation with IAA (20μL, 1mol/L) for 60 min in the dark, the samples were digested with trypsin at 37°C for 12 h [17].

### LC-MS/MS analysis

Peptide mixtures were used for protein identification by nano-liquid chromatography coupled with MS. The LC-MS/MS system consisted of an Agilent 1100 quaternary HPLC (EASY-nLC1000) and a micrOTOF-Q II mass spectrometer (Bruker Daltonics, USA) with the application of a distal 180°C source temperature. A RP trapcolumn (Thermo EASY column SC200 150μm×100mm) was used for desalting, and a C18 reverse-phase column (Thermo EASY column SC001 traps 150μm × 20mm) was used for separation. Mobile phase A consisted of HPLC-grade water containing 0.1% formic acid (FA), and phase B consisted of 84% HPLC-grade acetonitrile (ACN) containing 0.1% FA. The analytical separation was run at a flow rate of 400 nl/min using a linear gradient of phase B as follows: 0–45% for 100 min, 45–100% for 8 min and 100% for 12 min. To reduce the technical variation the analyses were repeated three times [37]. Peptide spectral matches (PSMs) were validated using the percolator provided by the Proteome Discoverer software based on q-values at a 1% false discovery rate.

Normalization by a reference sample, also known as probabilistic quotient normalization, is a robust method to account for different dilution effects of biofluids. This method is based on the calculation of a most probable dilution factor (median) by looking at the distribution of the quotients of the amplitudes of a test spectrum by those of a reference spectrum [38].

### Bioinformatics analysis

For protein identification, the Mascot 2.1 program (Matrix Science, Boston, MA, USA) was used for fragmentation spectra research, which was searched against the human database. Two missed cleavages were permitted, and an error of 6 ppm or 20 ppm was allowed for full MS or MS/MS spectra study, respectively. Furthermore, we used the resulting MGF documents and Data Analysis 3.4 from Bruker Daltonics Software to detect the fold-changes of the protein levels of each group. The identified proteins were analyzed by GO categories using the Web Gene Ontology Annotation Plotting (WEGO, http://wego.genomics.org.cn/cgi-bin/wego/index.pl). Signaling pathway analysis was performed by Kyoto Encyclopedia of Genes and Genome (KEGG) database (http://www.genome.jp/kegg/pathway.html) [39].

Proteins used for validation were selected by molecular function and the biological process or pathway term in PANTHER.

### Quantitative real-time PCR

Total RNA was obtained using miRNeasy Mini Kits (Qiagen, Valencia, CA, USA) according to the manufacturer’s instructions. Subsequently, 500 ng of the RNA was reverse transcribed to cDNA using ReverTra Ace qPCR RT Kits(TOYOBO, Osaka, Japan), and quantitative real-time PCR was carried out with a 7900 HT Sequence Detection System (ABI, Foster City, CA, USA) using ABI Power SYBR Green PCR Master Mix. The raw data were normalized using the Quantile algorithm in GeneSpring 11.0 (Agilent Technologies, Santa Clara, CA, USA). The thermal cycling conditions were: 2 min at 95°C for initial denaturation, followed by 40 cycles of 15 sec at 95°C, 60 sec at 60°C for amplification, and 15 sec at 95°C, 15 sec at 60°C and 15 sec at 95°C for melting curve analysis [6]. Target gene primers are listed in Supplementary Table 4.

### Western blot

Western bloting was performed as described previously, with minor differences [6]. Briefly, proteins extracted from plasma, were separated using 12% SDS-PAGE gels and transferred to PVDF membranes (Millipore, Billerica, MA, USA). For detection, after incubation with primary antibodies overnight at 4°C, the membranes were washed three times with Tris-buffered saline containing Tween-20 (TBS-T), and incubated with horseradish peroxidase (HRP) - conjugated secondary antibodies (1:5000; Santa Cruz, Dallas, TX, USA) for 2 h at room temperature. Antibody binding was visualized using the Enhanced Chemi Luminescence Kit (ECL-Kit, Santa Cruz) after being washed in PBS-T. The primary antibodies used in this experiment included rabbit polyclonal anti-XRCC4 antibody (Abcam, Cambridge, MA, USA, diluted 1:1000), rabbit polyclonal anti-PCF11 antibody (Abcam, diluted 1:2000), rabbit polyclonal anti-SEMA4A antibody (Abcam, diluted 1:1000), rabbit polyclonal anti-ATP11A antibody (Santa Cruz, diluted 1:1000), and mouse monoclonal anti-β-actin antibody (Upstate, Lake Placid, NY, USA; diluted 1:2000).

Ten μg protein was loaded per lane of a 1-mm-thick mini polyacrylamide SDS-gel. The proteins were stained with Coomassie Blue as an internal control for normalization [40] Proteins were transferred to a NC membrane for 1h using Bio-rad Trans-Blot. After being blocked with 3% BSA for 30min, the target proteins were incubated with primary antibody and associated secondary to each blot with at least four, 3-minute wash steps.

### Statistical analysis

Each experiment was repeated independently at least three times. Data are expressed as the mean ± SD and differences between groups were evaluated with the Student t-test or Mann-Whitney U test. Values of *P*<0.05 were considered statistically significant.

## SUPPLEMENTARY MATERIALS TABLES




