# Supplementary material for: Characterization of plasma proteins in children of different Mycobacterium tuberculosis infection status using label-free quantitative proteomics
Source: Oncotarget. 2017 Sep 23;8(61):103290–301. doi: 10.18632/oncotarget.21179 (PMC5732728; doi:10.18632/oncotarget.21179)
Supplement: Supplementary file 1 [file oncotarget-08-103290-s001.pdf]

# Characterization of plasma proteins in children of different *Mycobacterium tuberculosis* infection status using label-free quantitative proteomics

## SUPPLEMENTARY MATERIALS

**Supplementary Table 1: Classification of up-regulated proteins identified following LC-MS/MS of TB different from LTBI fractions**

See Supplementary File 1

**Supplementary Table 2: Classification of down-regulated proteins identified following LC-MS/MS of TB different from LTBI fractions**

| WEGO | Category | No. | Gene |
|---|---|---|---|
| | extracellular region | 1 | LOX |
| | extracellular region part | 1 | LOX |
| | cell | 6 | INPP5B, high similar to TJP2, JADE1, TSSK4, LOX, ZNF281 |
| | cell part | 6 | INPP5B, high similar to TJP2, JADE1, TSSK4, LOX, ZNF281 |
| | membrane-enclosed lumen | 2 | JADE1, ZNF281 |
| Cellular Component | macromolecular complex | 1 | JADE1 |
| | organelle | 5 | INPP5B, JADE1, TSSK4, ZNF281, LOX |
| | organelle part | 3 | INPP5B, ZNF281, JADE1 |
| | developmental process | 4 | ZNF281, TSSK4, INPP5B, LOX, |
| | reproduction | 2 | INPP5B, TSSK4 |
| | cellular component biogenesis | 1 | LOX |
| | cellular component organization | 2 | LOX, JADE1 |
| | metabolic process | 5 | ZNF281, INPP5B, TSSK4, JADE1, LOX |
| | death | 1 | JADE1 |
| | reproductive process | 2 | INPP5B, TSSK4 |
| | pigmentation | 4 | JADE1, INPP5B, ZNF281, TSSK4 |
| | locomotion | 1 | INPP5B |
| Biological Process | localization | 1 | INPP5B |
| | response to stimulus | 2 | LOX, JADE1 |
| | growth | 1 | JADE1 |
| | cellular process | 5 | JADE1, INPP5B, ZNF281, TSSK4, LOX, |
| | multicellular organismal process | 5 | JADE1, TSSK4, LOX, ZNF281, INPP5B |
| | anatomical structure formation | 1 | LOX |
| | biological regulation | 4 | TSSK4, JADE1, INPP5B, ZNF281, |
| | transcription regulator activity | 1 | ZNF281 |
| Molecular Function | enzyme regulator activity | 1 | INPP5B, |
| | catalytic activity | 3 | INPP5B, TSSK4, LOX, |
| | binding | 6 | ZSWIM8, TSSK4, ZNF281, INPP5B, LOX, JADE1 |

**Supplementary Table 3: KEGG enrichment analysis of the identified proteins**

| #Term | Database | ID | Input number | Background number | *P*-Value | Input |
|---|---|---|---|---|---|---|
| mRNA surveillance pathway | KEGG PATHWAY | hsa03015 | 2 | 124 | 0.018 | Q9H307\|O94913 |
| Non-homologous end-joining | KEGG PATHWAY | hsa03450 | 1 | 20 | 0.034 | Q13426 |
| Spliceosome | KEGG PATHWAY | hsa03040 | 2 | 190 | 0.040 | O75533\|P09651 |
| One carbon pool by folate | KEGG PATHWAY | hsa00670 | 1 | 30 | 0.049 | P11586 |
| Vitamin digestion and absorption | KEGG PATHWAY | hsa04977 | 1 | 32 | 0.053 | P04114 |
| Glycosphingolipid biosynthesis - lacto and neolacto series | KEGG PATHWAY | hsa00601 | 1 | 37 | 0.061 | Q11206 |
| Fat digestion and absorption | KEGG PATHWAY | hsa04975 | 1 | 57 | 0.092 | P04114 |
| Arachidonic acid metabolism | KEGG PATHWAY | hsa00590 | 1 | 80 | 0.125 | P20813 |
| Retinol metabolism | KEGG PATHWAY | hsa00830 | 1 | 91 | 0.141 | P20813 |
| Inositol phosphate metabolism | KEGG PATHWAY | hsa00562 | 1 | 107 | 0.164 | P32019 |
| Drug metabolism - cytochrome P450 | KEGG PATHWAY | hsa00982 | 1 | 112 | 0.171 | P20813 |
| Metabolism of xenobiotics by cytochrome P450 | KEGG PATHWAY | hsa00980 | 1 | 115 | 0.175 | P20813 |
| Protein digestion and absorption | KEGG PATHWAY | hsa04974 | 1 | 123 | 0.186 | Q5TAT6 |
| Phosphatidylinositol signaling system | KEGG PATHWAY | hsa04070 | 1 | 153 | 0.225 | P32019 |
| RNA transport | KEGG PATHWAY | hsa03013 | 1 | 220 | 0.307 | Q9H307 |
| Oxytocin signaling pathway | KEGG PATHWAY | hsa04921 | 1 | 239 | 0.329 | P30559 |
| Purine metabolism | KEGG PATHWAY | hsa00230 | 1 | 250 | 0.341 | Q9UIJ7 |
| Protein processing in endoplasmic reticulum | KEGG PATHWAY | hsa04141 | 1 | 260 | 0.352 | Q96KC8 |
| Metabolic pathways | KEGG PATHWAY | hsa01100 | 4 | 1832 | 0.360 | P20813\|P32019\|P11586\|Q11206 |
| Axon guidance | KEGG PATHWAY | hsa04360 | 1 | 268 | 0.360 | Q9H3S1 |
| Herpes simplex infection | KEGG PATHWAY | hsa05168 | 1 | 273 | 0.366 | P51610 |
| Calcium signaling pathway | KEGG PATHWAY | hsa04020 | 1 | 276 | 0.369 | P30559 |
| cAMP signaling pathway | KEGG PATHWAY | hsa04024 | 1 | 323 | 0.417 | P30559 |
| Neuroactive ligand-receptor interaction | KEGG PATHWAY | hsa04080 | 1 | 392 | 0.480 | P30559 |

**Supplementary Table 4: Target gene primers of quantitative real-time PCR**

| Primer Name | Sequence (5' to 3') |
|---|---|
| ATP11A-F | GGCCAACAGCAACAGAGAGAGT |
| ATP11A-R | GGCTGCTGGAAGTCTGAGAAA |
| PCF11-F | GCTGCTAAAGAAAAAGAGTTCCAAAG |
| PCF11-R | TTCATCCCAGTATTGTTCAAATTGTT |
| XRCC4-F | TGATGAGGAAAGTGAAAACCAAAC |
| XRCC4-R | GGTGCAATATCAGTGACATCAAGAC |
| SEMA4A-F | CCAGTCTCCCAAGGAATGCA |
| SEMA4A-R | GGCCTGTGCCTAGAGTTTAAGC |
| Gapdh-F | TGACTTCAACAGCGACACCCA |
| Gapdh-R | CACCCTGTTGCTGTAGCCAAA |
